# Engineered *Saccharomyces cerevisiae* for the Enhancement of Glucosamine Accumulation by the Consumption of Glucose and Ammonium Based on Synthetic Biological Pathways

**DOI:** 10.3390/foods14162783

**Published:** 2025-08-10

**Authors:** Peizhou Yang, Mingsi Ke, Jiaqi Feng, Zhi Zheng, Shaotong Jiang

**Affiliations:** School of Food and Biological Engineering, Anhui Province Key Laboratory of Agricultural Products Modern Processing, Hefei University of Technology, Hefei 230601, China; mingsike2002@163.com (M.K.); fengjqi@126.com (J.F.); zhengzhi@hfut.edu.cn (Z.Z.); jiangshaotong@163.com (S.J.)

**Keywords:** *Saccharomyces cerevisiae*, glucosamine production, CRISPR-Cas9 approach, transcriptomics analysis, ammonium sulphate

## Abstract

Glucosamine (GlcN) is a high-value compound with significant health applications. GlcN is widely used in the food and health industry as a food additive or functional food. The development of a green, efficient, and safe method for GlcN production is of great significance due to the complexity of traditional production methods, environmental pollution, and sensitization of raw materials. In this study, *Saccharomyces cerevisiae* genes *PFK1*, *PDB1*, *GNA1*, *ISR1*, and *PCM1* were knocked out using the Clustered Regularly Interspaced Short Palindromic Repeats Cas9 (CRISPR-Cas9) method. In addition, three key enzyme genes, glucosamine-6-phosphate deaminase *GlmD*, glucosamine-6-phosphate phosphatase *GlmP*, and ammonium transporter *AMT1*, were introduced to construct engineered strains for GlcN synthesis in the presence of high-concentration inorganic ammonium ions. The results indicated that *S. cerevisiae* HPG5 with *GlmD*, *GlmP*, and *AMT1* integration and simultaneous deletion of *PFK1*, *PDB1*, *GNA1*, *PCM1*, and *ISR1* achieved the highest GlcN yield (1.95 ± 0.02 g/L) during fermentation with 10 g/L (NH_4_)_2_SO_4_, which was 2.47-fold higher than the control. The conversion rate of glucose to GlcN in HPG5 was 9.75% in liquid YPD medium containing 20 g/L of glucose and 10 g/L of (NH_4_)_2_SO_4_. Thus, the results indicated that *S. cerevisiae* HPG5 could effectively produce GlcN in the presence of high-concentration ammonium sulphate. This study provides a promising alternative, *S. cerevisiae* HPG5, for GlcN production.

## 1. Introduction

Glucosamine (GlcN) is an amino sugar that provides a building block for crucial biological molecules with the primary function of glycosaminoglycans biosynthesis, such as hyaluronic acid, chondroitin sulfate, and keratan sulfate [1]. In addition, GlcN plays a vital role in maintaining joint health, cushioning, and flexibility [2]. GlcN was generally produced through the chemical separation and hydrolysis approach in previous production with high energy use and serious environmental pollution [3]. In recent years, the production of GlcN through microbial fermentation has rapidly developed. Compared with chemical decomposition methods, microbial fermentation requires lower costs and a wider range of raw material sources [4]. In addition, microbial fermentation is carried out under mild conditions, usually at room temperature, atmospheric pressure, and near neutral pH, which can avoid the hazards of extreme conditions such as high temperature, strong acid, and strong alkali [5].

*S. cerevisiae* is a commonly used host for biomanufacturing due to its robustness and regulatory acceptance [6]. However, native *S. cerevisiae* cannot directly produce GlcN due to the absence of key enzymes involved in GlcN synthesis [7]. In yeast metabolism, glucosamine-6-phosphate deaminase (GlmD) and glucosamine-6-phosphate phosphatase (GlmP) play key roles in the synthesis of GlcN [8,9]. In addition, ammonium transporter (AMT1) functions to enhance ammonium uptake and assimilation in yeast [10]. AMT1 integration facilitates the efficient transport of extracellular ammonium ions into yeast cells [11]. Theoretically, the integration of the above three genes is conducive to the accumulation and secretion of GlcN, thereby promoting the efficient synthesis of GlcN. Further, multiple *S*. *cerevisiae* genes are associated with GlcN accumulation as follows: (1) *PFK1* deletion reduces glycolytic flux downstream of fructose-6-phosphate and enhances substrate availability for GlcN production by minimizing competition from glycolysis [12]; (2) *PDB1* deletion alleviates metabolic competition for carbon sources, indirectly supporting GlcN synthesis by reducing energy-intensive processes [13]; (3) *GNA1* deletion blocks the conversion of GlcN-6-P to GlcNAc-6-P and increases intracellular accumulation of GlcN-6-P [14]. Heterologous *GlmP* subsequently dephosphorylates GlcN-6-P to release free GlcN [15]; (4) *PCM1* deletion reduces flux toward glycogen and UDP-glucose-dependent pathways (e.g., cell wall polysaccharides) and increases G-6-P availability, which is isomerized to F-6-P, further feeding HBP for GlcN synthesis [16]; and (5) *ISR1* deletion disrupts sphingolipid synthesis and frees up resources (e.g., ATP and NADPH) for GlcN biosynthesis [17].

**Figure 1:**
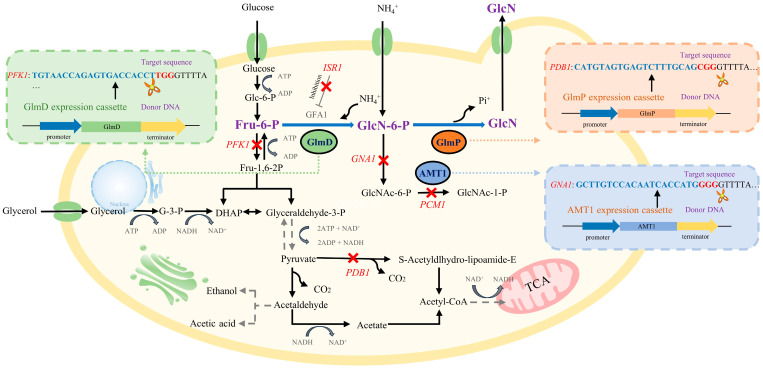
Modification strategy of *S. cerevisiae* for GlcN synthesis based on the synthetic biology pathway. Note: This strategy comprehensively considered several factors, including key enzymes in GlcN synthesis (GlmD and GlmP), metabolic flux improvement of GlcN synthesis (*PFK1*, *GNA1*, *PCM1*, and *ISR1* deletion), transport efficiency enhancement of ammonium ions (AMT1), and tolerance improvement (*PDB1* deletion), to achieve efficient intracellular synthesis of GlcN in engineered *S. cerevisiae*.

In this study, engineered *S. cerevisiae* strains were constructed by deleting *PFK1*, *PDB1*, *GNA1*, *PCM1*, and *ISR1* and introducing *GlmD*, *GlmP*, and *AMT1* based on the principles of synthetic biology (Figure 1). This study developed an engineered *S. cerevisiae* with high-yield GlcN in the presence of glucose and ammonium sulphate by modifying the GlcN synthesis pathway.

## 2. Materials and Methods

### 2.1. Strains, Plasmids, Antibiotics, and Instruments

A haploid *S. cerevisiae* strain was used to construct the engineered strains for GlcN production. *S. cerevisiae* was cultured at 30 °C in YPD medium prepared with 1% yeast extract (*w/v*), 2% peptone (*w/v*), and 2% glucose (*w/v*). *E. coli* DH5α for plasmid replication was cultured in LB medium prepared with 5 g/L yeast extract (*w/v*), 10 g/L tryptone (*w/v*), and 10 g/L NaCl (*w/v*). Plasmids Cas9-NAT and gRNA-trp-HyB contained nourseothricin and hygromycin B resistance genes, respectively. The High-Fidelity PCR Master Mix, NAT, HyB, plasmids, and ssDNA were from Sangon Biotech (Shanghai, China). All chemical reagents were analytical grade. UV spectrophotometry (UV-1780, Shimadzu Corporation, Kyoto, Japan) and high-performance liquid chromatography (HPLC) (Agilent 1260, Agilent Technologies Inc., Santa Clara, CA, USA) were used.

### 2.2. Determination of gRNA Sequences of Target Gene

Guide RNA (gRNA) sequences from *S. cerevisiae PFK1*, *PDB1*, *GNA1*, *PCM1*, and *ISR1* were determined to knock out the target genes using programming tools from the online website https://chopchop.cbu.uib.no/, accessed on 8 August 2025. The primer sequences containing 23-bp gRNA sequences for the amplification of gRNA vectors (*PFK1*-gRNA, *PDB1*-gRNA, *GNA1*-gRNA, *PCM1*-gRNA, and *ISR1*-gRNA) are listed in Table 1. The 46-bp primer sequences included 23-bp gRNA and another 23-bp plasmid template sequence. The PCR reaction system consisted of 2 × Phusion master mix (12.5 μL), upstream primer (1 μL), downstream primer (1 μL), template DNA (0.5 μL), and ultra-pure water (10 μL). The PCR amplification conditions were as follows: preheat to 95 °C for 30 s, denaturation at 95 °C for 15 s, annealing at 56 °C for 15 s, extend at 72 °C for 4 min and 30 s for 30 cycles, and final extension at 72 °C for 5 min. The gRNA-trp-HyB vector after amplification was used to screen the putative transformants.

### 2.3. S. cerevisiae Genetic Transformation and Engineered Strain Construction

The LiAc/SSDNA approach was used to transform exogenous genes and plasmid vectors into yeast cells under PEG mediation [18]. Both plasmids Cas9-NAT and gRNA-trp-HyB were transformed into yeast cells on screening media containing antibiotics [19]. The transformation sampling systems were as follows: total volume of 360 µL, 50% (*w/v*) PEG 3350 (240 µL), 1 M LiAc (36 µL), ssDNA (50 µL), template DNA (2 µL), and deionized water (32 µL). The final transformants were screened on solid YPD medium containing 100 μg/mL of hygromycin and 80 μg/mL of nourseothricin antibiotics. The engineered yeasts needed to discard the plasmids containing the antibiotic resistance genes inside the cells to achieve the deletion of the genes. The putative transformants were confirmed using gene sequencing after amplification of the targeted genes.

### 2.4. Comparison of GlcN Yields from Different Engineered S. cerevisiae Strains

The colonies of wild-type *S. cerevisiae* and engineered strains HPG1, HPG2, HPG3, HPG4, and HPG5 were cultured in 10 mL of liquid YPD medium at 30 °C for 48 h at a shaking speed of 180 rpm. The fermentation broth was injected into a 250 mL Erlenmeyer flask containing 150 mL of YPD medium. After fermentation at 30 °C for 48 h with a shaking speed of 180 rpm, the fermentation solution was extracted to determine the yields of GlcN from different *S. cerevisiae* strains.

### 2.5. GlcN Yields from S. cerevisiae HPG2 and HPG5 During Fermentation

The cell colonies of *S. cerevisiae* HPG2, HPG5, and wild-type strains were cultured in 10 mL of YPD liquid medium at 30 °C for 48 h. Then, the cells were added to 150 mL of YPD liquid medium and fermented at 30 °C with a shaking speed of 180 rpm. The yields of GlcN were determined during 72 h of fermentation using YPD liquid medium containing 20 g/L of glucose. The conversion rate of glucose to GlcN was calculated by dividing the GlcN content by 20 g/L of glucose.

### 2.6. Cell Proliferation, GlcN Yield, and Glucose Consumption of S. cerevisiae HPG5

The *S. cerevisiae* HPG5 colony was cultivated and proliferated according to the above steps. The fermentation broth of *S. cerevisiae* HPG5 was extracted for the determination of cell concentration, GlcN yield, and glucose content. The cell proliferation of *S. cerevisiae* HPG5 was determined during 72 h of fermentation by measuring OD values at the wavelength of 600 nm. In addition, the glucose consumption of *S. cerevisiae* HPG5 was also investigated during 72 h of fermentation by determining the glucose content. Further, the GlcN yield of *S. cerevisiae* HPG5 was determined by measuring GlcN concentrations in YPD fermentation broth containing 20 g/L of glucose.

### 2.7. Effect of (NH_4_)_2_SO_4_ on the Growth of HPG2 and HPG5 on Solid YPD Media

To investigate the stress tolerance of *S. cerevisiae* HPG2 and HPG5, 2 µL of solution sample after four dilutions (10^−1^, 10^−2^, 10^−3^, and 10^−4^) was dropped on YPD solid media containing 10, 20, and 30 g/L of (NH_4_)_2_SO_4_. After cultivation at 30 °C for 48 h, the growth of each colony was observed under the same viewing field. In addition, the control group was observed on YPD medium without the addition of (NH_4_)_2_SO_4_.

### 2.8. GlcN Yield and Glucose Consumption in the Presence of 10 g/L of (NH_4_)_2_SO_4_

The GlcN yields and glucose concentrations of *S. cerevisiae* HPG2 and HPG5 were compared during 72 h of fermentation. The YPD fermentation medium contained 10 g/L of (NH_4_)_2_SO_4_ and 20 g/L of glucose. In addition, the conversion rate of GlcN from glucose was calculated by dividing the GlcN content by 20 g/L of glucose.

### 2.9. Measurement of GlcN, Glucose, and Ethanol Contents

The content of GlcN was detected using the HPLC approach with the following parameters: Waters Alliance e2695, Waters 2489 UV detector, an NH_2_ column (4.6 mm × 250 mm, 5 μm), a detection wavelength of 195 nm, a mobile phase of acetonitrile with phosphate buffer at a flow rate of 1.5 mL/min, and an injection volume of 10 μL [20]. The contents of glucose and ethanol were also determined using the HPLC approach with a mobile phase of 0.01 mol/L H_2_SO_4_, a flow rate of 0.8 mL/min, column temperature 50 °C, Waters 1525 binary HPLC pump, 2410 refractive detector, and Shodex SH1011 chromatographic column [21].

### 2.10. Cell Viability Determination and Genetic Stability Analysis

Loeffler’s methylene blue solution staining method was used to identify living and dead yeast cells according to the following steps [22]. (1) Yeast suspension was prepared by diluting the yeast sample in sterile water to a slightly turbid suspension (OD_600_ of 0.5); (2) Stain and sample were mixed in a 1:1 ratio by combining 100 µL of yeast suspension and 100 µL of Loeffler’s methylene blue, with incubation for 3 min at room temperature; (3) Microscopic examination was performed by observing under 400-times magnification. The percentage of viability (%) was calculated by dividing the number of unstained cells by the number of total cells, multiplied by 100. The genetic stability of *S. cerevisiae* HPG5 was analyzed by the determination of GlcN yields after 1st, 10th, 20th, 30th, 40th, and 50th generations of *S. cerevisiae* HPG5.

### 2.11. Transcriptomics Analysis

The wild-type and engineered *S. cerevisiae* strains were cultured in liquid YPD medium at 30 °C and a shaking speed of 180 rpm. The cells were harvested by centrifugation at 1000× *g* rpm for 5 min after fermentation for 48 h. The total RNA was isolated using the FastPure Universal Total RNA Isolation Kit. The concentration and integrity of RNA were determined using a Qubit2.0 fluorometer and nucleic acid gel electrophoresis. The total RNA was further treated by Sangon Biotech Company (Shanghai, China). The construction of the RNA sequence library and gene sequencing were performed using the Illumina Hiseq 2500 platform. The raw data were processed using FastQC. Differentially expressed genes (DEGs) were identified using GEO2R. Then, the functions of DEGs were analyzed through Gene Ontology (GO) and Kyoto Encyclopedia of Genes and Genomes (KEGG) pathway enrichment [23].

### 2.12. Data Analysis

Data are presented as the mean ± standard deviation (SD) from triplicate experiments. Significance was assessed using Origin 2024 software.

## 3. Results

### 3.1. Engineered S. cerevisiae Construction Using the CRISPR-Cas9 Method

**Figure 2:**
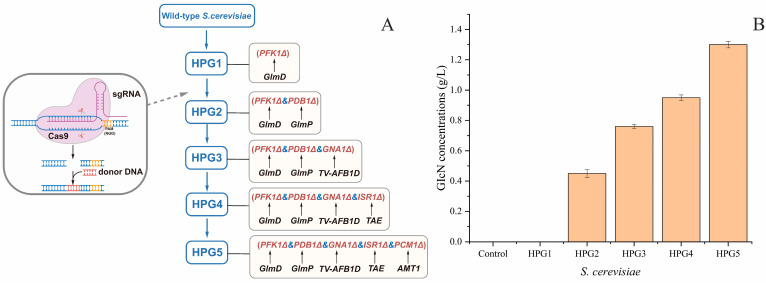
Pathway of engineered *S. cerevisiae* construction and GlcN yield comparison of different yeasts. (**A**) Sequential construction of multiple engineered yeasts using the CRISPR-Cas9 approach. (**B**) Comparison of GlcN yields from different yeasts.

The engineered *S. cerevisiae* strains were constructed through the integration of exogenous genes and the deletion of complete pathways. The five engineered *S. cerevisiae* strains after deleting different gene combinations were named *S. cerevisiae* HPG1, HPG2, HPG3, HPG4, and HPG5 (Figure 2A). The putative *S. cerevisiae* transformants were screened on solid YPD plates containing hygromycin and nourseothricin antibiotics. *S. cerevisiae* transformants were identified through PCR amplification of target genes. The sizes of the insertion genes were consistent with the target genes. All of the putative engineering strains were confirmed through gene sequencing.

### 3.2. Comparison of GlcN Yields from Five Engineered S. cerevisiae Strains

The GlcN yields from the five engineered *S. cerevisiae* strains were determined using the wild-type strain as the control (Figure 2B). The results indicated that the highest yield of GlcN was from *S. cerevisiae* HPG5 (1.3 ± 0.02 g/L), which was 2.89-times higher than that of HPG2 (0.45 ± 0.03 g/L), 1.71-times higher than that of HPG3 (0.76 ± 0.01 g/L), and 1.37-times higher than that of HPG4 (0.95 ± 0.02 g/L). Meanwhile, the concentrations of GlcN from HPG1 and the control could not be measured. Thus, the integration of both *GlmD* and *GlmP* caused GlcN production in *S. cerevisiae*. *S. cerevisiae* HPG5 achieved a high efficiency of GlcN production among the tested strains.

### 3.3. Effect of Gene Deletion on GlcN Production

**Figure 3:**
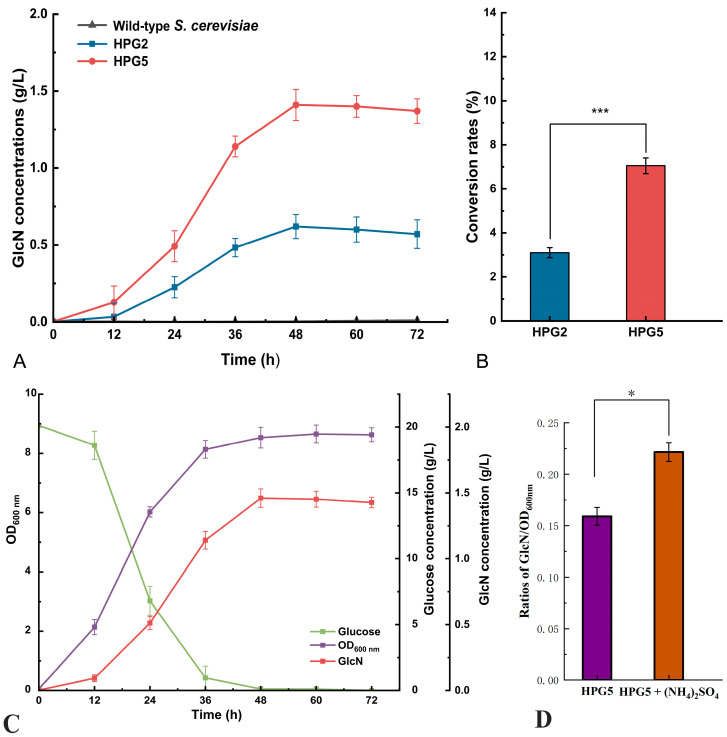
GlcN concentrations, glucose concentrations, and OD_600nm_ values. (**A**,**B**) GlcN concentrations and glucose to GlcN conversion rates of *S. cerevisiae* HPG2 and HPG5, respectively. (**C**) OD_600nm_ values, glucose, and GlcN concentrations of *S. cerevisiae* HPG5 during fermentation. (**D**) Ratios of GlcN and OD_600nm_ from HPG5 and HPG5 with the addition of 10 g/L (NH_4_)_2_SO_4_. * and *** represent significant differences at the levels of 0.05 (*p* < 0.05) and 0.001 (*p* < 0.001), respectively.

The GlcN concentrations of *S. cerevisiae* HPG2 and HPG5 were investigated using the wild-type strain as the control. The highest GlcN concentrations from *S. cerevisiae* HPG2 and HPG5 were 0.62 ± 0.01 g/L and 1.41 ± 0.02 g/L, respectively, after fermentation for 48 h, with no GlcN production from the wild-type strain (Figure 3A). The GlcN conversion rates of glucose from *S. cerevisiae* HPG2 and HPG5 were 3.1% and 7.05%, respectively (Figure 3B). The GlcN production of *S. cerevisiae* HPG5 was 2.27-fold higher than that of *S. cerevisiae* HPG2. The GlcN concentration of *S. cerevisiae* HPG5 decreased to 1.37 ± 0.02 g/L after fermentation for 72. Therefore, *S. cerevisiae* HPG5 after gene modification remarkably increased GlcN production by reconstructing the synthesis pathway of GlcN in yeast cells.

### 3.4. GlcN Production, Glucose Consumption, and Cell Growth of S. cerevisiae HPG5

The OD_600 nm_ values, GlcN, and glucose contents of *S. cerevisiae* HPG5 were determined during liquid fermentation for 72 h (Figure 3C). The content of glucose decreased to 0.1 g/L after a fermentation for 48 h. The OD_600 nm_ value of *S. cerevisiae* HPG5 was 8.65 when yeast cell proliferation reached the plateau phase after 48 h of fermentation. The GlcN content of 1.46 ± 0.03 g/L in *S. cerevisiae* HPG5 was the highest during fermentation for 48 h. The accumulation of GlcN during liquid fermentation was accompanied by increasing cell growth and glucose consumption. In addition, the ratios of GlcN and OD_600nm_ from HPG5 and HPG5 with the addition of 10 g/L (NH_4_)_2_SO_4_ in the fermentation broth were compared (Figure 3D). The ratio from HPG5 with the addition of 10 g/L (NH_4_)_2_SO_4_ (0.22 ± 0.01 g/L/OD) was 1.38-times higher than that from HPG5 (0.16 ± 0.01 g/L/OD). Therefore, *S. cerevisiae* HPG5 had high expression efficiency of GlcN under liquid fermentation conditions using glucose as the fermentation substrate.

### 3.5. Effect of (NH_4_)_2_SO_4_ on the Proliferation of S. cerevisiae HPG5

**Figure 4:**
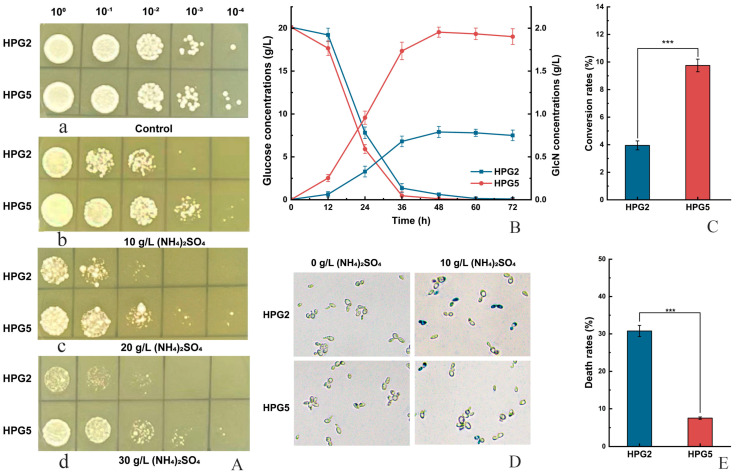
Tolerance test of (NH_4_)_2_SO_4_, GlcN yield comparison, and cell viability determination. (**A**) Tolerance test at the different concentrations of (NH_4_)_2_SO_4_ of *S. cerevisiae* HPG2 and HPG5 on solid media. Note: a, b, c, and d represent colony growth on solid media containing 0 (the control), 10, 20, and 30 g/L of (NH_4_)_2_SO_4_ with gradient dilution 10^4^, respectively. (**B**) GlcN production and glucose contents of *S. cerevisiae* HPG2 and *S. cerevisiae* HPG5 in media containing 10 g/L (NH_4_)_2_SO_4_. (**C**) Conversion rates of glucose and GlcN in *S. cerevisiae* HPG2 and HPG5. (**D**) Cell viability determination of *S. cerevisiae* HPG2 and HPG5 using the merocyanine staining method. (**E**) Death rates of *S. cerevisiae* HPG2 and HPG5 after treatment for 12 h in media containing 10 g/L (NH_4_)_2_SO_4_. *** represents significant differences at the level of 0.001 (*p* < 0.001).

The colony growth of *S. cerevisiae* HPG5 was investigated on solid YPD media containing different concentrations of (NH_4_)_2_SO_4_ compared to *S. cerevisiae* HPG2. The results demonstrated that both strains could grow on the solid media with no significant difference (Figure 4(A-a)). A cluster of *S. cerevisiae* HPG5 colonies grew on the solid media containing 10 g/L (NH_4_)_2_SO_4_ after dilution 10^3^ times, with only two *S. cerevisiae* HPG2 colonies (Figure 4(A-b)). Further, both 20 g/L and 30 g/L concentrations of (NH_4_)_2_SO_4_ inhibited the cell proliferation of *S. cerevisiae* HPG5 and HPG2 (Figure 4(A-c,d)). The colony numbers of *S. cerevisiae* HPG5 were remarkably higher than those of *S. cerevisiae* HPG2 after dilution 10^2^ and 10^3^ times. Therefore, *S. cerevisiae* HPG5 exhibited stronger tolerance to ammonium ions than *S. cerevisiae* HPG2 at high concentrations of (NH_4_)_2_SO_4_.

### 3.6. Effect of (NH_4_)_2_SO_4_ on GlcN Production and Cell Viability of S. cerevisiae HPG5 in Submerged Fermentation

The GlcN concentrations of *S. cerevisiae* HPG5 were measured in liquid media containing 10 g/L (NH_4_)_2_SO_4_. The GlcN concentration of *S. cerevisiae* HPG5 (1.95 ± 0.02 g/L) was 2.47-fold higher than that of *S. cerevisiae* HPG2 (0.79 ± 0.01 g/L) after fermentation for 48 h (Figure 4B). The conversion rates of glucose to GlcN of *S. cerevisiae* HPG2 and HPG5 were 3.95% and 9.75%, respectively (Figure 4C). Furthermore, the viabilities of yeast cells were investigated using the cell staining approach. *S. cerevisiae* HPG5 and HPG2 cells were stained in liquid fermentation without (NH_4_)_2_SO_4_. The colony numbers of stained *S. cerevisiae* HPG5 cells were lower than those of HPG2 after the addition of 10 g/L of (NH_4_)_2_SO_4_ in fermentation media. The death rate of *S. cerevisiae* HPG5 (7.5%) accounted for 24.4% compared with HPG2 (30.7%) according to the statistics of the number of stained cells (Figure 4D,E). Thus, *S. cerevisiae* HPG5 improved GlcN production using glucose as the substrate in the presence of high concentrations of ammonium sulfate.

### 3.7. Comparison of GlcN Yields from Different Engineered Strains

The comparison of GlcN yields from HPG2 and HPG5 under different fermentation conditions was investigated (Table 2). The results indicated that HPG5 produced a higher GlcN yield than HPG2 using YPD as the liquid fermentation broth. In addition, the GlcN yields of HPG2 and HPG5 after 48 h of fermentation were higher than those after 72 h of fermentation. Furthermore, the addition of 10 g/L (NH_4_)_2_SO_4_ resulted in the increase in GlcN yields of HPG5 and HPG2 from 1.41 ± 0.02 g/L to 1.95 ± 0.02 g/L and 0.62 ± 0.01 g/L to 0.79 ± 0.01 g/L, respectively. Thus, the addition of 10 g/L (NH_4_)_2_SO_4_ remarkably increased the GlcN yields of HPG5 and HPG2.

### 3.8. Genetic Stability of S. cerevisiae HPG5 for the Production of GlcN

**Figure 5:**
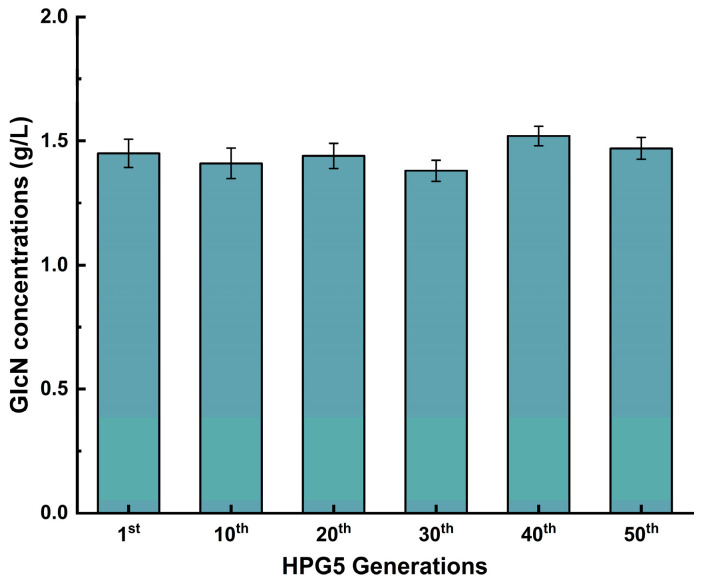
Effect of generations on the GlcN production of *S. cerevisiae* HPG5.

*S. cerevisiae* HPG5 was constructed by deleting *PFK1*, *PDB1*, *GNA1*, *PCM1*, and *ISR1* and integrating *GlmD*, *GlmP*, and *AMT1* to produce GlcN based on synthetic biology principles and metabolic engineering strategies. The genetic stability was analyzed by determining the content of GlcN via dozens of generations (Figure 5). The GlcN contents were 1.45 ± 0.04, 1.41 ± 0.02, 1.44 ± 0.04, 1.38 ± 0.04, 1.52 ± 0.03, and 1.47 ± 0.03 g/L after the 1st, 10th, 20th, 30th, 40th, and 50th generations. No significant difference existed between the data above. Therefore, *S. cerevisiae* HPG5 maintained genetic stability in producing GlcN after several passages. This study provides a solid foundation for further utilization for intracellular GlcN production.

### 3.9. The Distribution of DEGs

**Figure 6:**
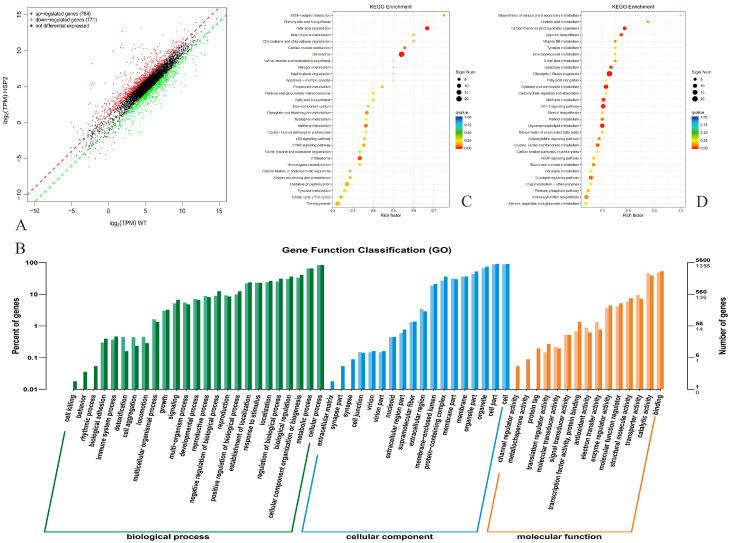
Transcriptomics of *S. cerevisiae* HPG5. Note: (**A**,**B**) Distribution and GO annotation of DEGs, respectively. (**C**,**D**) KEGG enrichment analysis.

The transcriptomic data of engineered HPG5 and the control strain EHS2 in Supplementary File S1 were compared to explore the effect of gene deletion on gene expression after constructing a cDNA library for sequencing analysis (Figure 6). The results demonstrated that a total of 1555 significantly differentially expressed genes were identified in engineered HPG5. According to the analysis of log2 (TPM) values, 784 up-regulated genes and 771 down-regulated genes were identified (Figure 6A). The number of inhibitory genes in genetically modified HPG5 exceeded the number of stimulating genes.

### 3.10. GO Annotation of DEGs

The GO classifications of engineered HPG5 and the control strain included the categories of molecular function, cellular component, and biological process based on the transcriptomics sequencing data (Figure 6B). In terms of biological processes, the DEGs of HPG5 were mainly enriched in cell aggregation, proliferation, multicellular biological processes, and multi-organism processes. In terms of molecular function, the DEGs of HPG5 were mainly enriched in the extracellular region, membrane part, and membrane. In terms of molecular function, the DEGs of HPG5 were mainly enriched in molecular transduction activity, transporter activity, catalytic activity, etc. Therefore, genetic modification of HPG5 resulted in significant differences in gene expression compared to the control strain.

### 3.11. KEGG Enrichment Analysis

The KEGG pathway enrichment analysis results are demonstrated in Figure 6C,D. The upregulated DEGs in the 30 enrichment pathways of HPG5 were mainly enriched in fatty acid degradation, peroxisomes, proteasomes, glyoxylate and dicarboxylic acid metabolism, and oxidative phosphorylation processes. The highest enrichment factor of 0.67 in the upregulated DEGs was found in the fatty acid degradation metabolic pathway, which indicated the enhanced ability of HPG5 to degrade fatty acids. Fatty acid degradation produced more acetyl CoA, which enters the tricarboxylic acid cycle to generate more energy to maintain normal growth and metabolism. The upregulation of proteasome related genes was related to the deletion of the *PDB1* gene and the expression of AMT1. The activity increase of proteasome efficiently degraded misfolded or useless proteins, maintained protein homeostasis in cells, facilitated the normal growth of yeast, and helped yeast to cope with environmental stress induced by high-concentration ammonium ions. The downregulated DEGs were mainly enriched in carbon metabolism pathways such as glycolysis and partial sugar metabolism, cellular metabolism pathway, and partial amino acid metabolism. Therefore, the simultaneous deletion of *PFK1*, *PDB1*, *GNA1*, *ISR1*, and *PCM1* genes in HPG5 caused the upregulation of gene expression in cellular metabolic pathways such as fatty acid degradation and proteasome.

## 4. Discussion

The introduction of *GlmD* and *GlmP* into metabolic pathways represents a non-canonical approach to glucosamine synthesis, which has been primarily explored in metabolic engineering for microbial production. GlmD possesses a native function of catalyzing the deamination of glucosamine-6-phosphate (GlcN-6-P) to fructose-6-phosphate and NH_3_ in catabolic pathways (e.g., *E. coli* hexosamine degradation) and an engineering twist of synthesizing GlcN-6-P from fructose-6-P and NH_3_ in the presence of high ammonia (NH_3_) concentrations [24]. This bypasses the canonical GlcN-6-P synthase (GlmS), which requires glutamine as a costly nitrogen donor [25]. In addition, GlmP possesses a function of hydrolyzing GlcN-6-P to GlcN and inorganic phosphate [26]. This step is essential to dephosphorylate GlcN-6-P for extracellular export or accumulation. The rationale of GlmD and GlmP has three advantages: (1) cost reduction by replacing glutamine-dependent GlmS with NH_3_-driven synthesis (NH_3_ is cheaper than glutamine); (2) regulatory bypass by avoiding feedback inhibition of GlmS by UDP-GlcNAc (a downstream product); and (3) pathway simplification by creating a shorter route: fructose-6-P + NH_3_ → [GlmD] → GlcN-6-P → [GlmP] → glucosamine.

Transcriptomic analysis revealed critical metabolic adaptations in HPG5 that underpinned its enhanced phenotype. The upregulation of fatty acid degradation pathways (enrichment factor: 0.67) generates abundant acetyl-CoA, which fuels the TCA cycle to produce ATP and NADPH. This energy surplus compensates for *PFK1* deletion-impaired glycolysis and powers GlcN biosynthesis under ammonium stress. Proteasome pathway upregulation correlates with *PDB1* deletion and *AMT1* integration. Enhanced proteasomal activity degrades misfolded proteins caused by high ammonium influx, maintaining proteostasis and freeing resources (e.g., ATP and amino acids) for GlcN synthesis. This explains HPG5′s superior viability (7.5% vs. 30.7% cell death rate in HPG2) in the presence of 10 g/L (NH_4_)_2_SO_4_. Conversely, the downregulation of glycolysis and sugar metabolism aligns with *PFK1* knockout, redirecting carbon flux from competing pathways toward fructose-6-phosphate, the precursor for the heterologous GlmD/GlmP pathway.

Zhang et al. introduced *GlmD* from *E. coli* and GlmP from *S. cerevisiae* into *B. subtilis* with a GlcN yield of 3.2 g/L using glucose and (NH_4_)_2_SO_4_ [27]. This study introduced *Pyrococcus furiosus* genes *GlmD*, *GlmP*, and ammonium transporter *AMT1* using the CRISPR-Cas9 approach to construct engineered *S. cerevisiae* strains with a GlcN yield of 1.95 ± 0.02 g/L. The GlmD/GlmP pathway offers a viable, nitrogen source-flexible alternative for glucosamine synthesis, particularly in engineered bacteria. While yields are competitive, several metabolic challenges remain unresolved. First, product inhibition of GlmD by accumulated GlcN likely constrains titers in yeast at higher concentrations. Second, native metabolic flux toward UDP-GlcNAc synthesis competes with the heterologous pathway for GlcN-6-P, limiting GlcN secretion. Third, despite AMT1-enhanced ammonium uptake, high (NH_4_)_2_SO_4_ concentrations (≥20 g/L) induces cellular stress, resulting in reduced proliferation and viability.

Future efforts should prioritize overcoming these barriers. Enzyme engineering of GlmD for reduced product inhibition or improved kinetics under industrial conditions could boost flux. Dynamic flux control strategies may better balance carbon allocation between glycolysis, UDP-GlcNAc synthesis, and GlcN production. Additionally, further pathway balancing (e.g., downregulating UDP-GlcNAc transferases) could minimize competitive losses. Addressing ammonium toxicity via co-expression of stress-responsive chaperones or alternative nitrogen assimilation routes would enhance robustness.

The GlmD/GlmP pathway holds promise for sustainable GlcN production, but scale-up challenges (e.g., NH_3_ volatility, kinetic inefficiencies, and cellular stress) warrant deeper investigation. In this study, the transcriptomic data already reveal adaptive rewiring in HPG5 (e.g., upregulated proteasomes/fatty acid degradation), suggesting inherent compensatory mechanisms that could be leveraged in future designs.

## 5. Conclusions

This study constructed an engineered *Saccharomyces cerevisiae* strain (HPG5) using the CRISPR-Cas9 system by integrating heterologous genes *GlmD*, *GlmP*, and *AMT1* and simultaneously deleting endogenous genes *PFK1*, *PDB1*, *GNA1*, *PCM1*, and *ISR1.* Under fermentation conditions of YPD medium with 20 g/L glucose and 10 g/L (NH_4_)_2_SO_4_, HPG5 achieved a GlcN titer of 1.95 ± 0.02 g/L and a glucose-to-GlcN conversion rate of 9.75%. Transcriptomic analysis of HPG5 revealed 1555 significantly differentially expressed genes (784 upregulated and 771 downregulated) compared to the control strain. Key upregulated pathways included fatty acid degradation and the proteasome system, suggesting metabolic adaptations to channel resources (e.g., acetyl-CoA and energy) toward GlcN synthesis and ammonium ion stress. Downregulated pathways included glycolysis and sugar metabolism, consistent with the deletion of *PFK1*. This work provides a genetically stable and efficient engineered *S. cerevisiae* HPG5 platform for the microbial production of glucosamine, offering a potential alternative to traditional shellfish extraction. In addition, HPG5′s robustness under high ammonium conditions, coupled with its genetic stability over 50 generations, positions it as a promising candidate for scalable bioprocess integration. Future optimization of fed-batch fermentation and downstream processing could further enhance its industrial viability.

## Figures and Tables

**Table 1:** Primers for gRNA vector construction and donor DNA synthesis.

Primers	Sequences (5′→3′)	Genes
*PFK1*-gRNA-F	TGTAACCAGAGTGACCACCTTGGGTTTTAGAGCTAGAAATAGCAAG	*PFK1*-gRNA
*PFK1*-gRNA-R	CCAAGGTGGTCACTCTGGTTACAGATCATTTATCTTTCACTGCGGA	
*PDB1*-gRNA-F	TTCCATTAGAGCATTCAAAGCGGGTTTTAGAGCTAGAAATAGCAAG	*PDB1-gRNA*
*PDB1*-gRNA-R	CCGCTTTGAATGCTCTAATGGAAGATCATTTATCTTTCACTGCGGA	
*GNA1*-gRNA-F	GCTTGTCCACAATCACCATGGGGGTTTTAGAGCTAGAAATAGCAAG	*GNA1*-gRNA
*GNA1*-gRNA-R	CCCCATGGTGATTGTGGACAAGCGATCATTTATCTTTCACTGCGGA	
*PCM1*-gRNA-F	ACTAACCAGAGATTACCCAAGGGGTTTTAGAGCTAGAAATAGCAAG	*PCM1*-gRNA
*PCM1*-gRNA-R	CCCTTGGGTAATCTCTGGTTAGTGATCATTTATCTTTCACTGCGGA	
*ISR1*-gRNA-F	GCCGACAGCTAAATTCAGTGAGGGTTTTAGAGCTAGAAATAGCAAG	*ISR1*-gRNA
*ISR1*-gRNA-R	CCTCACTGAATTTAGCTGTCGGCGATCATTTATCTTTCACTGCGGA	
TAE-F/R	GAGACTTTTCAAAGGGT/GATCTGGATTTTAGTACTGG	2848 bp
AMT1-ADH1-F/R	GTTGATTGTATGCTTGGTATAGC/CCGGTAGAGGTGTGGTCAATAAG	2401 bp
pEGFP-GlmD-F/R	CGGGGTCATTAGTTCATAGCCC/GCCCGCTCCTTTCGCTTTCTTC	2977 bp
pEGFP-FE838-F/R	ACGGGGTCATTAGTTCATAGCC/CCGCTCCTTTCGCTTTCTTCCC	2622 bp
TV-AFB1D-F/R	ATGGCTCGCGCGAAGTACTC/TTAAAGCTTCCGCTCTATGAA	2091 bp

Note: Primers of *PFK1*-gRNA-F/*PFK1*-gRNA-R, *PDB1*-gRNA-F/*PDB1*-gRNA-R, *GNA1*-gRNA-F/*GNA1*-gRNA-R, *PCM1*-gRNA-F/*PCM1*-gRNA-R, and *ISR1*-gRNA-F/*ISR1*-gRNA-R were used to amplify the gRNA vectors of *PFK1*-gRNA, *PDB1-gRNA, GNA1*-gRNA, *PCM1*-gRNA, and *ISR1*-gRNA, respectively. In addition, primers of TAE-F/R, AMT1-ADH1-F/R, pEGFP-GlmD-F/R, pEGFP-FE838-F/R, and TV-AFB1D-F/R were used to amplify *TAE*, *AMT1*, *GlmD*, *FE838*, and *AFB1D*, respectively.

**Table 2:** GlcN yields in engineered strains under different conditions in this study.

Strain	Fermentation Media, Time, and Genetic Characteristics	GlcN Yield (g/L)
HPG2	YPD, 72 h, *PFK1ΔPDB1Δ* with *GlmD*/*GlmP* integration	0.45 ± 0.03
HPG5	YPD, 72 h, *PFK1ΔPDB1ΔGNA1ΔISR1ΔPCM1Δ* with *GlmD*/*GlmP/AMT1* integration	1.3 ± 0.02
HPG2	YPD, 48 h, *PFK1ΔPDB1Δ* with *GlmD*/*GlmP* integration	0.62 ± 0.01
HPG5	YPD, 48 h, *PFK1ΔPDB1ΔGNA1ΔISR1ΔPCM1Δ* with *GlmD*/*GlmP/AMT1* integration	1.41 ± 0.02
HPG2	YPD + 10 g/L (NH_4_)_2_SO_4_, 48 h, *PFK1ΔPDB1Δ* with *Glm*/*GlmP* integration	0.79 ± 0.01
HPG5	YPD + 10 g/L (NH_4_)_2_SO_4_, 48 h, *PFK1ΔPDB1ΔGNA1ΔISR1ΔPCM1Δ* with *GlmD*/*GlmP/AMT1* integration	1.95 ± 0.02

## Data Availability

The raw data supporting the conclusions of this article will be provided by the authors.

## Supplementary Materials

The following supporting information can be downloaded at: https://www.mdpi.com/article/10.3390/foods14162783/s1. Supplementary File S1. *S. cerevisiae* DEGs of *S. cerevisiae* HPG5 based on the comparative transcriptomics.
